# Supplementary figures and images for: A Comparative Study of Serum Angiogenic Biomarkers in Cirrhosis and Hepatocellular Carcinoma
Source: Cancers (Basel). 2021 Dec 21;14(1):11. doi: 10.3390/cancers14010011 (PMC8750498; doi:10.3390/cancers14010011)

Supplementary Information

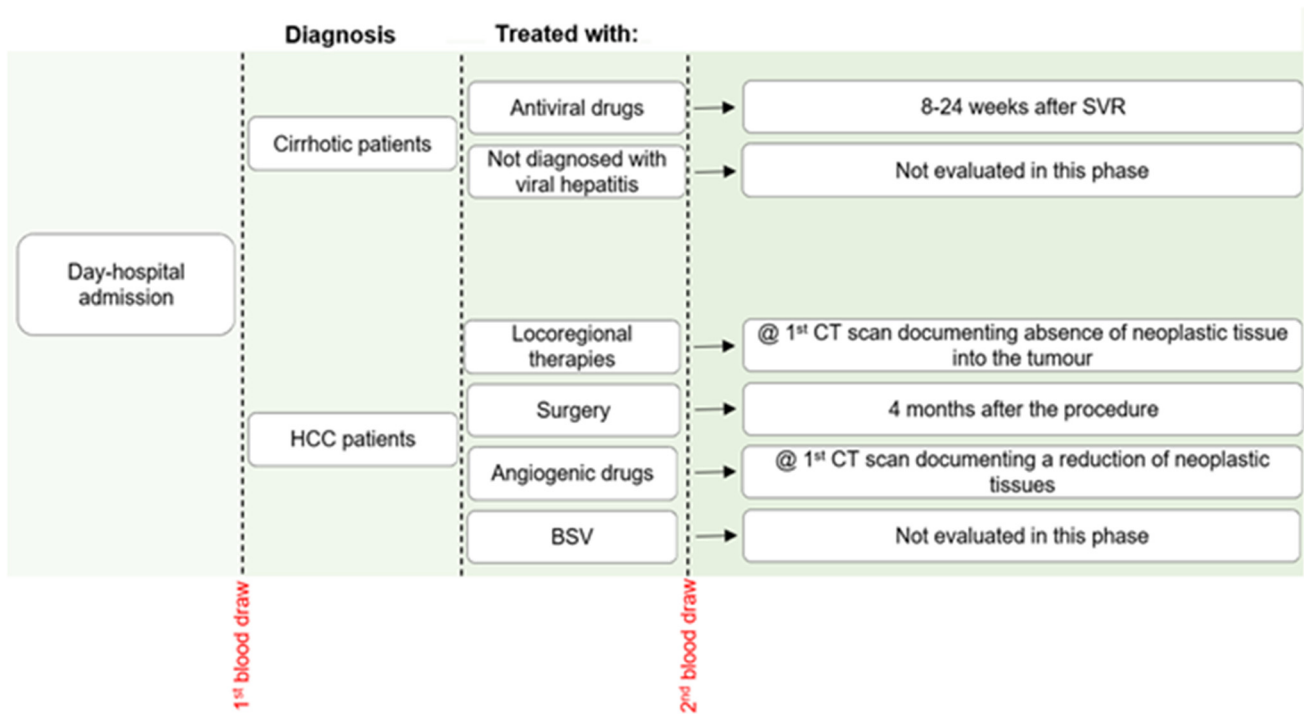

Figure S1. schematic workflow.

Supplement: Supplementary file 1 [file cancers-14-00011-s001.zip › cancers-1522584-supplementary.pdf]
